# Evolutionary Tree for All Bumblebee Species World-Wide Estimated by Combining Information from Fast-Evolving Genes, Slow-Evolving Genes, and Genomic Data (Apidae, *Bombus*)

**DOI:** 10.3390/insects17060540

**Published:** 2026-05-22

**Authors:** Paul H. Williams, Pedro Alonso-Alonso, Marina Arbetman, Elaine Françoso, Guillaume Ghisbain, Jiaxing Huang, Michael C. Orr, Zong-Xin Ren, Martin Streinzer, Chawatat Thanoosing, Rémy Vandame, Madeleine Waite, Selina Brace

**Affiliations:** 1 Natural History Museum, Cromwell Road, London SW7 5BD, UK; madeleinewaite02@gmail.com (M.W.); s.brace@nhm.ac.uk (S.B.); 2 Department of Animal Ecology and Tropical Biology, Biocenter, University of Würzburg, 97070 Würzburg, Germany; pedro.alonso-alonso@uni-wuerzburg.de; 3 Grupo de Ecología de la Polinización (EcoPol), INIBIOMA (Comahue-CONICET), Quintral 1250, Bariloche 8400, Río Negro, Argentina; marbetman@comahue-conicet.gob.ar; 4 Department of Biological Sciences, Royal Holloway, University of London, Surrey TW20 0EX, UK; francoso.e@gmail.com; 5 Laboratory of Zoology, Research Institute for Bioscience, University of Mons, Place du Parc 20, 7000 Mons, Belgium; guillaume.ghisbain@umons.ac.be; 6 Institute of Apicultural Research, Chinese Academy of Agricultural Sciences, 2 Yuanmingyuan West Road, Haidian, Beijing 100093, China; huangjiaxing@caas.cn; 7 Institute of Zoology, Chinese Academy of Sciences, 1 Beichen West Road, Chaoyang, Beijing 100101, China; michael.christopher.orr@gmail.com; 8 Staatliches Museum für Naturkunde Stuttgart, 70191 Stuttgart, Germany; 9 Kunming Institute of Botany, Chinese Academy of Sciences, 132 Lanhei Road, Kunming 650201, China; renzongxin@mail.kib.ac.cn; 10 University of Vienna, Universitätsring 1, 1010 Vienna, Austria; martin.streinzer@univie.ac.at; 11 Bee and Spider Research Unit, Faculty of Science, Chulalongkorn University, Bangkok 10330, Thailand; chawatat.t@chula.ac.th; 12 Departamento Agricultura Sociedad y Ambiente, El Colegio de la Frontera Sur (ECOSUR), San Cristóbal de Las Casas 29290, Chiapas, Mexico; remy@ecosur.mx

**Keywords:** bayesian analysis, bumblebee, COI barcode, diversification, evolutionary tree, phylogeny, trait

## Abstract

Evolutionary trees are of central importance in biology to develop explanatory frameworks for many kinds of studies, including studies of behaviour, ecology, and conservation. Since the last major estimate of evolutionary relationships among many bumblebee species two decades ago, there have been revisions to their taxonomy, descriptions of new species, and sequencing of many rarer species. By combining: (1) earlier data mostly from slow-evolving nuclear genes as a backbone tree; with (2) new data from fast-evolving mitochondrial COI barcodes to resolve more of the twigs on the tree, including sequences obtained from rare old specimens with ancient DNA techniques; as well as (3) the results from broader genomic data for resolving deep relationships, we make a Bayesian estimate of evolutionary relationships with BEAST 2 among all 289 published and accepted extant bumblebee species, an increase of more than 29% of the species on the previous largest tree. The new tree will serve as a framework for future comparative studies that should enable broader insights into the evolution and ecology of all bumblebees. We illustrate this with an analysis of the evolution of some male morphological characters related to male mate-searching behaviour. We also present a novel map of spatial variation in net diversification rates among bumblebee species world-wide, which indicates an especially rapid net diversification within the more recent Mesoamerican and South American faunas.

## Simple Summary

To estimate the evolutionary tree for all of the world’s bumblebee species, we combine (1) earlier data for mostly slow-evolving nuclear genes, with (2) new data for a fast-evolving mitochondrial gene, and (3) results from a study of genomic data. The resulting tree provides an up-to-date starting point for comparative studies of all bumblebees.

## 1. Introduction

Evolutionary trees are essential as a framework for studies in comparative biology. For bumblebees (tribe Bombini, genus *Bombus*) these studies have focused especially on morphology [1,2], behaviour [3,4,5], ecology [1,4,5], biogeography [1,6,7,8], and conservation [8,9,10]. Here we present an improved estimate of the evolutionary tree for bumblebees that includes all currently accepted, extant species to help support these studies. It is important when interpreting all of these studies to appreciate that the rate of discovery of bumblebee species new to science has now greatly slowed since the middle of the twentieth century, so that most bumblebee species may already be known [11]. This adds to their value as a model group.

When recent comparative studies of bumblebees have referred to an evolutionary tree they have most often used the tree estimate from Cameron et al. [12] (or its ‘nearly identical’ re-run in Hines [6]). This tree was estimated using Bayesian methods (MrBayes) from sequence data for segments of four nuclear genes (for long-wavelength rhodopsin copy 1 (‘opsin’); elongation factor-1 alpha F2 (‘Ef-1α’); arginine kinase (‘ArgK’); and phosphoenolpyruvate carboxykinase (‘PEPCK’); and one mitochondrial gene, a 16S-length sequence coding for ribosomal RNA (‘16S’). The title of the Cameron et al. paper is ‘*A comprehensive phylogeny of the bumble bees*’ and while it was the best dataset available at the time, it covers just 71% of the currently recognised extant species of bumblebees. The shortfall has arisen partly because a few species have been described more recently, but mainly because many species have always been very difficult to find, to identify, and to sequence (see below).

To extend the Cameron et al. tree [12] to cover all currently recognised bumblebee species, there are several advantages in supplementing the earlier analysis with DNA-sequence data from ‘barcodes’ from a standard region (COI-5P) of the COI gene. These are relatively fast-evolving mitochondrial DNA sequences [13,14], so that they are especially well suited to resolving relationships among the most closely-related missing ‘twigs’ of the tree [15]. COI barcodes are also inexpensive to acquire and already widely available for many species [16]. With improving access and sequencing methods they can now be obtained even for the rarest species from older specimens (e.g., [17,18,19]).

The problem with any tree that estimates evolutionary relationships for species that is based on only a few genes (whether nuclear or mitochondrial) is that individual genes are not always reliable for resolving the species’ tree. This is because of discordances that are known to exist among trees for individual genes [20] and which have been demonstrated for bumblebees [6,12]. Individual slow-evolving nuclear genes may also contain little or no information relating to many of the divergences, making these difficult to resolve, especially if changes took place within a short time interval that was long ago, a problem that is best resolved by using a large sample of nuclear genes [21]. Fortunately, this information is now available for bumblebees from the genomic data assembled by Sun et al. [22]. Of the total of around 16,000 genes in the bumblebee genome, Sun et al. sampled parts of ca 10,000 genes to produce an estimate of relationships among representatives of all 15 bumblebee subgenera (the major evolutionary groups of species [23]). The Sun et al. tree therefore represents the best available estimate of the deeper relationships among global bumblebee diversity. However, it includes only 19 species.

We seek to obtain an improved estimate of the evolutionary tree of relationships for all extant bumblebee species by bringing together the strengths of all three approaches. We combine: (1) the five-gene, mostly nuclear, data from Cameron et al. [12] to build a backbone for the tree [13,24]; adding (2) mitochondrial barcode data to further resolve the terminal relationships for the remaining 29% of bumblebee species; and (3) ensure that the tree has the best current estimates of deep-branch relationships as supported from the extensive cross-genome sampling of Sun et al. [22]. An important contribution here for some globally very rare species is the use of ancient DNA techniques in obtaining sequences from old specimens. Our resulting tree represents the culmination of our current knowledge of bumblebee evolutionary relationships and serves as the basis for downstream comparative studies on the biology of this fascinating group.

## 2. Materials and Methods

### 2.1. Species’ List and Species’ Identification

A fundamental part of estimating complete evolutionary trees that often goes unrecognized is: (1) the need for a complete global species’ list for a group; and (crucially) (2) the ability to identify all of the species correctly. This is not straightforward because ideas of the nature of species are far from universally agreed, with changing views through time [25,26]. This results in species’ lists for bumblebees that are not only contentious at any one time, but have a long history of change [27]. Even if conceptual issues with the species’ problem in general may never be completely resolved [25], nonetheless a research programme that seeks to maximise consistency in the interpretation of bumblebee species would clearly make comparisons easier [11,28,29,30,31]. Therefore estimating an evolutionary tree demands a single list of species that ideally requires a choice of a consistent species’ concept or definition.

What are species? Bumblebees are especially problematic for taxonomy because they are very variable, so that while nearly 3000 taxa have been named from this variation, they have been interpreted as having only around one tenth of that number of species [11]. After more than half a century of emphasis on a *Reproductive Isolation Species’ Concept* (known more widely and less precisely as the ‘biological’ species concept) that did not lead to a discrete testable criterion [25], there has recently been a quiet revolution among specialist taxonomists. For the last 15 years the most common practice in recognising species for bumblebees has been to apply the definition of species as *Evolutionarily Independent Lineages* (EILs) [32]. The practical implementation of this idea has been to assess candidates with an ‘integrative’ test, which requires corroboration from at least two test criteria that each supports the presence of separate species [33]. These criteria ideally represent successive steps on the road to speciation, and the breadth of accepted criteria leads to this idea also being seen as a *Unified Concept* [32]. In its application to bumblebees, the best criteria with theoretically-defined thresholds most often include: (1) support for separate species’ gene coalescents (for a pattern of evolutionarily independent lineages [34]); and (2) coincidence of discontinuous step-like changes in patterns of variation between populations for (ideally) multiple morphological differences (which can be interpreted as evidence for a lack of interbreeding between those populations [35]). The EIL definition has been accepted as the most appropriate for taxonomy [26].

**Figure 1:**
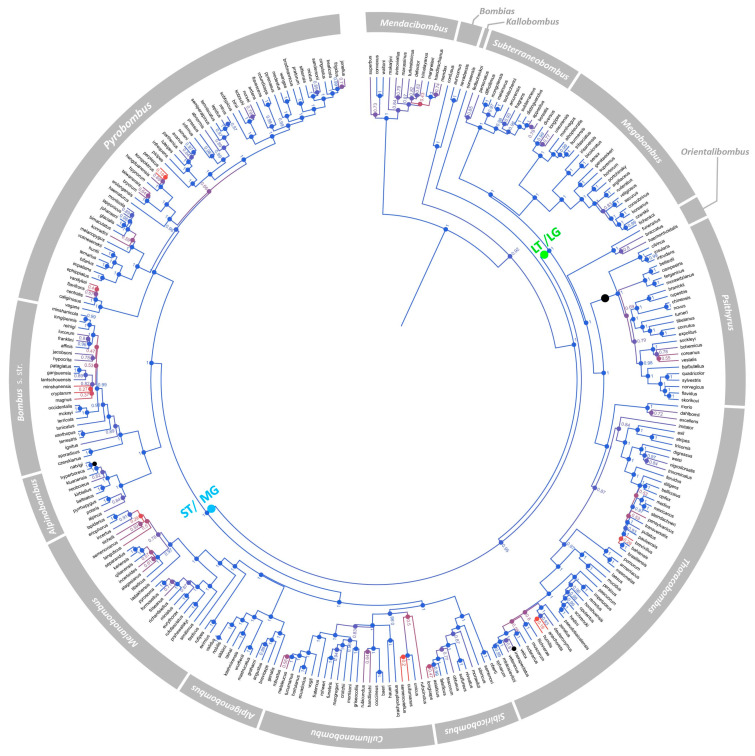
Evolutionary tree for all 289 extant accepted bumblebee species from 93 million MCMC generations (post convergence) by BEAST 2 for two mitochondrial genes, four nuclear genes, and genomic-supported group priors, with outgroups removed. Bayesian posterior support values for each node are shown with node and descendant branch colours indicating values from blue for high values to red for low values. Selected groups are indicated with larger spots: pale blue LT/LG for long-tongued *Lowland Grassland* species; pale green ST/MG for short-tongued *Montane Grassland* species; black spots for parasitic or ‘cuckoo’ species that lack workers, including the subgenus *Psithyrus* and *B. inexspectatus*, *B. hyperboreus*, *B. natvigi*. Subgenera are named in the outer grey ring. Taxon names not appearing in this figure are interpreted as synonyms (see text).

Most bumblebees world-wide have been revised using the EIL definition of species within subgenera, within species groups, and for some individual species [17,35,36,37,38,39,40,41,42,43,44,45,46,47,48,49,50,51,52,53,54,55]. Among many examples, some previously proposed ‘species’ (e.g., *B. applanatus*, *B. melanopoda* [7,19]) have been shown to be no longer supported as separate species (all prospective species have been reviewed from morphological and genetic evidence). For the species’ identification for the sample of *B. brevivillus* that was sequenced, a species for which there are differences in interpretation [7,56,57], the identification from Santos-Junior et al. [58] is accepted. The list of world bumblebee species accepted here includes 289 species (see Figure 1, listed in Supplementary Table S1: List of extant accepted bumblebee species world-wide), of which 84 species (29%) can now be added to the Cameron et al. [12] tree.

### 2.2. Gene Sequences

The sequences for the four nuclear genes and for the mitochondrial 16S gene used in the Cameron et al. [12] tree are all published on GenBank. The changing taxonomy and nomenclature of the species they listed has been tracked and revised for Supplementary Table S1: List of extant accepted bumblebee species world-wide. The sequence alignments used are based on Cameron et al. [12]. For a few other species, data can now be added for the same gene segments [17,41,58,59]. This data set can then be used to provide a ‘backbone’ from which to extend the tree [13,24]. This approach is feasible only because the five-gene coverage for the backbone is both extensive (70% of species) and evenly spread across the tree, including representatives of all subgenera and of all major species’ groups within subgenera.

Onto this backbone of mainly nuclear genes, all of the remaining species can be added by using data for the mitochondrial cytochrome *c* oxidase subunit I (COI) gene. A standard DNA segment of 657 nucleotides, the COI barcode (the COI-5P region) is widely used in species’ identification because it is rapidly-evolving [13,14] and thereby can inform even close relationships among species [15]. This gene has the additional advantage that many bumblebee COI-barcode sequences are already available from published sources [7,17,37,38,39,40,41,42,44,45,47,48,56,59]. Consequently a few more sequences were obtained from the public databases of BOLD (boldsystems.org) and GenBank (https://www.ncbi.nlm.nih.gov/genbank/ (accessed on 19 April 2026)), but only when their species’ identity could be confirmed (these databases contain substantial numbers of identification errors). Other sequences were acquired by our network of collaborators. These COI-barcode sequences had been obtained by Sanger sequencing using standardised protocols [15].

For some especially rare, supposed species from remote and inaccessible areas, including *B. fedtschenkoi*, *B. kotzschi*, *B. melanopoda*, *B. novus*, *B. oberti*, *B. senex* and *B. variabilis*, recent specimens were unavailable for sequencing. However, in these cases it was possible for the NHMUK Ancient DNA Laboratory to obtain nearly complete COI-barcode sequences from older museum specimens. This ‘aDNA’ approach uses specialist laboratory methods to extract DNA from a single bee leg [60]. Libraries for sequencing were built following using AmpliTaq Gold polymerase for the PCR amplification with 16 PCR cycles [61]. Libraries were shotgun sequenced on an Illumina NovaSeq. DNA read data was bioinformatically processed using the same de novo approach [62] using MitoBim [63] with starting seed sequences (listed in Supplementary Table S2: List of the seed species and COI5P barcode sequences used to obtain target-taxon barcode sequences with MitoBim). COI barcodes were then extracted from the mitochondrial genome generated from each sample. No attempt has been made to include the likely extinct species *B. rubriventris* (probably extinct >190 years ago, known from only one specimen [51]), although the possibly extinct *B. franklini* [64] is included.

The aligned gene data span 5420 nucleotide positions with a maximum of data for any one species of 4584 nucleotide positions. Gene-sequence identifiers are given in Supplementary Table S1: List of extant accepted bumblebee species world-wide with sequence identifiers.

### 2.3. Genomic Information

The best estimates of deep-branch relationships currently available to us are expected to be supported from the cross-genome Illumina sequencing, which represents parts of approximately 10,000 genes [22]. Their results show groups of subgenera (resolving uncertainties from the Cameron et al. tree) that are asserted here on the tree *a priori* by using Bayesian priors for the groups: (1) *Subterraneobombus* + *Megabombus*; (2) *Orientalibombus* + *Thoracobombus* + *Psithyrus*; and (3) *Alpigenobombus* + *Melanobombus*.

### 2.4. Age Calibration

Age calibration is made here with a Bayesian prior based on the dated tree of Hines [6], which in turn used the Cameron et al. [12] tree in combination with molecular rates and point calibration from fossils from outside of the genus *Bombus*. Unfortunately, fossils inside the genus *Bombus* [65] are not informative for calibrating this tree, because these fossils are already consistent with the Hines date estimates. We follow the procedure of Hines [6] in fixing the date of the divergence from the most recent common ancestor within the genus *Bombus* at 34 Ma.

### 2.5. Evolutionary Tree Estimation

Trees were estimated from the concatenated six-gene dataset using the Bayesian procedure BEAST 2 (version 2.7.7 [66]). The best nucleotide-substitution model for the concatenated data set according to the Bayesian information criterion in MEGA6 (version 6.06 [67]) is the general time-reversible model with a gamma-frequency distribution of changes among sites plus some invariant sites (GTR + Γ + I). The XML settings are scripted for BEAST 2 using BEAUti (version 2.7.7 [66]): to include the site model with four gamma categories; a log-normal relaxed clock model; a calibrated Yule model; priors added to identify the outgroups (*Eulaema boliviensis*, *Geniotrigona thoracica*, and *Plebeia frontalis* from Cameron et al. [12]) and a monophyletic ingroup, the monophyletic groups identified from Sun et al., and the most recent common ancestor node date; with the MCMC set to 100 million generations to give a sample of 10,000 trees. Tracer (version 1.6.0 [68]) was used to examine the trace files to check whether convergence was obtained. TreeAnnotator (version 2.6.6) was used to find a maximum clade-credibility tree with common ancestor heights after convergence. Trees were drawn with FigTree (version 1.4.4, http://tree.bio.ed.ac.uk/software/figtree/ (accessed on 19 April 2026)) and Illustrator (Adobe, version 26.0.1).

### 2.6. Diversification

The simplest representation of diversification rates from the evolutionary tree is to count the number of lineages-through-time [69], for which we use the Phytools package (version 2.5-2 [70]) on the R software platform (version 4.5.2 [71]). Our data have the advantage in this context that they include all of the known species that we consider to be currently extant within the monophyletic group of bumblebees.

An unexplored aspect of net diversification rates has been the examination of their spatial pattern. We apply a measure of net diversification rate that is formulated here so that for each species (tree terminal) the path back to the tree root is traced and the inverse of the internode branch lengths is summed. This gives higher weight to those species with many short internodes (i.e., with rapid speciation without extinction) along this path. When the measure is summed for all species within an area’s fauna, the effect is to give high scores to those areas that currently have more species with rapid speciation in their past. However, this does not necessarily imply that this rapid speciation all took place within this area, although if bumblebees are less dispersive than some other groups [8], then this speciation is more likely to have been more closely associated with current distributions. This net diversification-rate measure has been included (as the function, inv_branch_dist()) as an extension of the Treestats package (version 1.71.10 [72]) on the R platform. These faunal scores for net diversification rates are plotted for every occupied grid cell on the map (using the Worldmap software, version 26.1.6 [73,74]). The net diversification-rate pattern can then be compared with a map for species richness (updated from [11]) by overlaying the two in different colours [75].

### 2.7. Character Evolution

Ancestral changes are mapped onto the tree for two morphological characters of males using the Mesquite software (version 4.02 [76]). Males are available for all species except for *B. tanguticus*. For a preliminary analysis, the single most likely distribution of changes in ancestral character states is mapped using stochastic character mapping [77,78]. These morphological characters are measures of sexual dimorphism that may show continuous variation (Figure 10 in [35], and [2]), but for a preliminary analysis thresholds can be applied to score the character states as categories. For male antennal length, the categorical states are defined as: (0) for antenna either not reaching beyond the wing bases (more similar to females); or (1) for antenna strongly elongated so that they reach back beyond the wing bases. For male eye size: (0) for eye similar in size to that of females; (1) for eye moderately or strongly enlarged by comparing eye breadth from the anterior aspect with ocello-ocular distance (as the threshold, species with only weakly enlarged male eye, e.g., *B. cullumanus*, are not included in this category).

## 3. Results

### 3.1. Dated Evolutionary Tree

The genetic data varied as expected, with parsimony-informative sites 49% for COI, 42% for 16S, and 27–29% for the nuclear genes.

**Figure 2:**
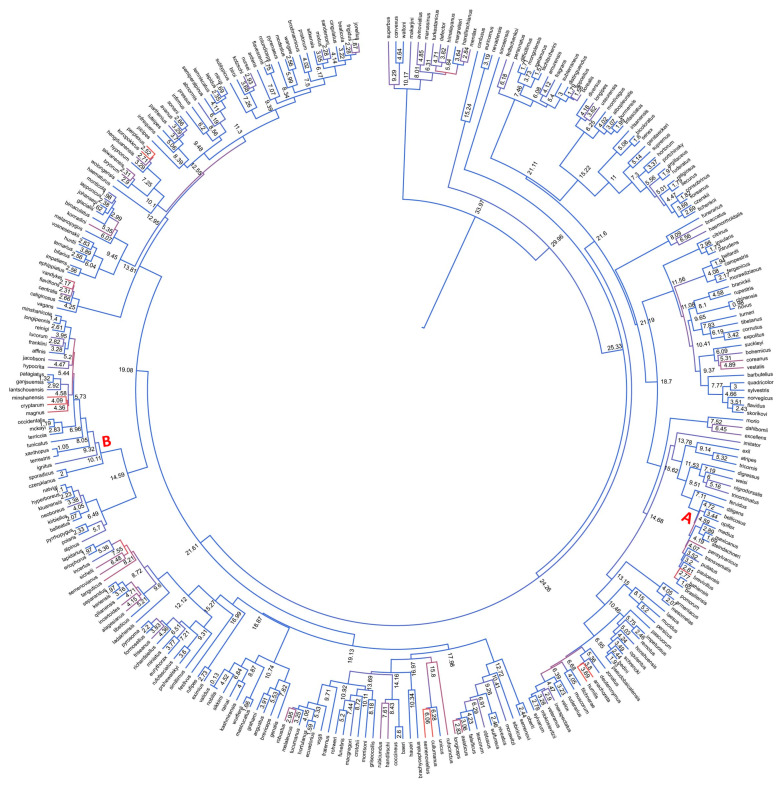
Evolutionary tree from Figure 1 (with branch colours for Bayesian posterior support values from Figure 1) with estimated node dates in Ma before the present. Two rapidly speciating groups are indicated with the letters in red A and B.

With 100 million MCMC generations, the TRACER results from BEAST 2 had converged on stable solutions by a burn-in of 7% of the generations. The post-burn-in effective sample sizes were high, with just one scoring <100. The great majority of nodes on the tree are strongly supported (Figure 1). The age of each node on the tree is estimated in Ma before the present (Figure 2).

### 3.2. Diversification

**Figure 3:**
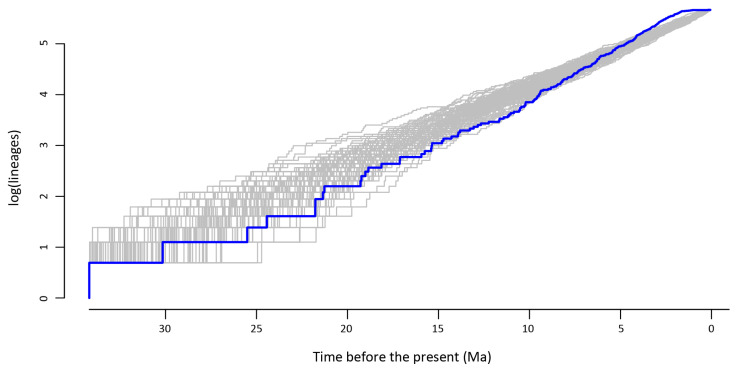
Number of bumblebee lineages through time from Figure 1 (in blue) compared with 100 simulations from a simple pure-birth process (in grey).

The observed number of bumblebee lineages through time is compared with simulations from a simple pure-birth model (of speciation without extinction) of lineage diversification (Figure 3), so that deviations from the observed number are usually expected to be due to extinctions. The observed number of lineages through time in Figure 2 does not yield a straight line (Figure 3, in blue), but a curve that initially deviates concavely below many of the null models (shown in grey) and finally deviates convexly above all of the null models.

**Figure 4:**
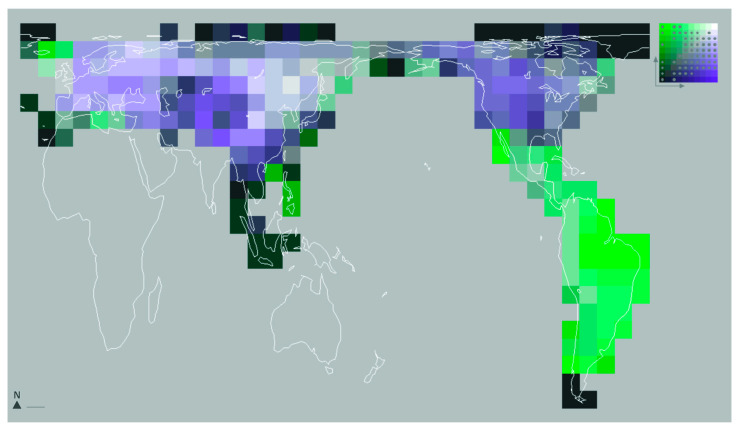
Comparison of species richness (in blue, or blue/purple on some devices) overlayed with speciation rates (in green: from summing the inverse of the internode-branch lengths on the root paths on the evolutionary tree to each species from Figure 2) mapped for bumblebee faunas on an equal-area grid (cylindrical equal-area projection, orthomorphic at 46° North and South of the equator, Antarctica not shown). Both colour axes are transformed to give near-uniform frequency distributions of scores among colour classes (see the colour-scale box, upper right: colour values represented for occupied cells on the map are indicated in the scale box with grey spots). Grid cells with high scores on both green and blue axes appear white, whereas cells with low scores on both axes appear black, while areas of intermediate and precisely covarying scores appear in intermediate shades of grey. By contrast, deviations from the overall positive relationship appear as increasingly saturated green or blue, showing an ‘excess’ of one score over the other. North pointer and scale bar representing 1100 km at the equator shown at lower left. For further explanation of the data see the text.

The spatial pattern in rates of net diversification (also assuming a pure-birth or speciation without extinction process as in Figure 3) is compared in Figure 4 with the map for species richness (the results of the diversification) for bumblebees. The most prominent feature of this map is that Central America, and more especially South America, is bright green (upper left of the colour key: low richness + high rates). This shows that while these areas have a lot fewer species than Asia, many of their species have more very short internodes that indicate periods in their histories of much more rapid speciation. Bright intense blue areas (lower right of the colour key: high richness + low diversification rates) are associated especially with the Himalaya, which shows many more species but with histories of more evenly long internodes indicating slower speciation. White and light grey or pale areas, especially in North-Eastern Asia, show a combination of both many species together with histories of relatively rapid speciation.

### 3.3. Character Evolution

**Figure 5:**
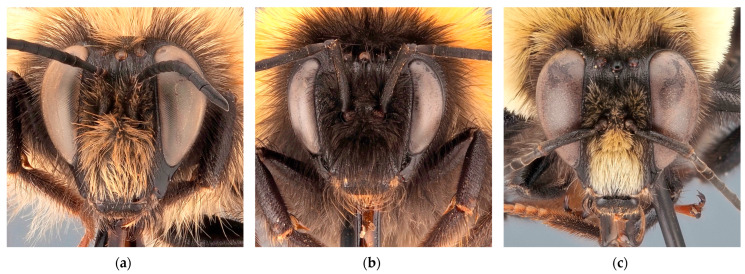
Anterior aspect of the heads of males of: (**a**) *B.* (*Mendacibombus*) *mendax*; (**b**) *B.* (*Bombus*) *terrestris*; and (**c**) *B. (Sibiricobombus) niveatus*. From the fit of the male eye-size character changes to the tree in Figure 6, the enlarged eye in (**a**) represents the ancestral enlarged state for the genus *Bombus*; the unenlarged eye similar in size to the female eye in (**b**) is a derived reduced state that is shared by most (83%) bumblebee species; and the secondarily enlarged eye in (**c**) is an evolutionary parallel with (**a**). Images by Paolo Rosa, ORBIT project, CC by 4.0.

**Figure 6:**
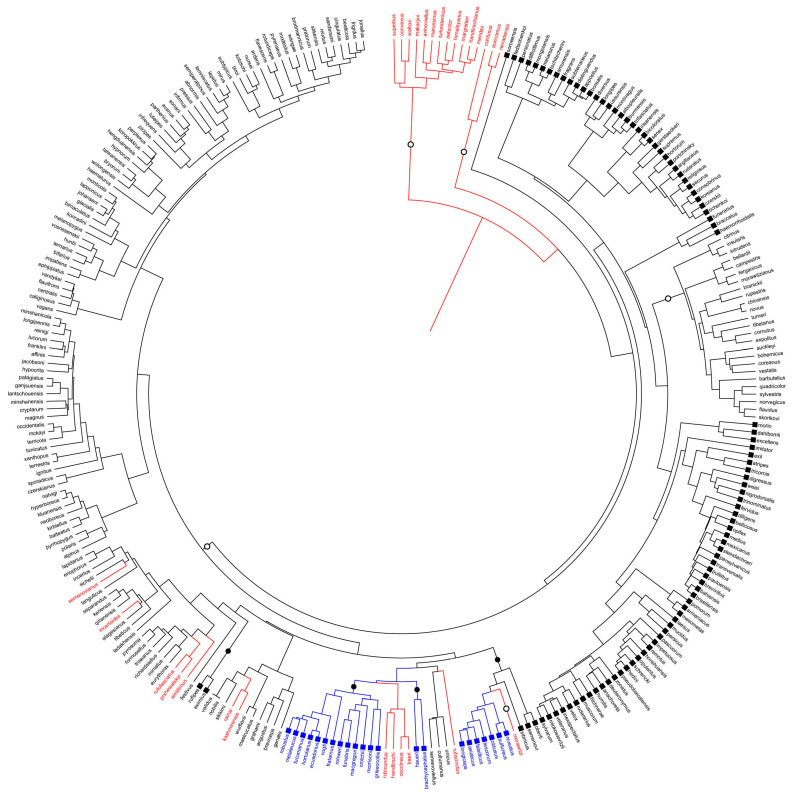
Evolutionary tree from Figure 1 with the most likely distribution on the tree of the ancestral character changes in male antennal length and male eye size fitted with stochastic character mapping. Most likely ancestral changes in male eye size are shown with changes in branch colour from red to black to red or blue (including reversals). Most likely changes in ancestral states of the antennal length are shown with spots of black (increases) or white (reversals) on branches and with squares on the terminals. Red branches indicate males combining enlarged eyes with short antennae, which is expected to indicate true territorial behaviour; the blue branches indicate males combining enlarged eyes with long antennae, which is expected to indicate scramble or ‘racing’ mate-searching behaviour; the black branches are expected to indicate male patrolling or nest-watching behaviours [35]. Taxon names not appearing in this figure are interpreted as synonyms (see text).

For the example of male character evolution, males of 40% bumblebee species have elongated antennae whereas males of only 17% species have moderately or strongly enlarged eyes (Figure 5). The distribution of ancestral changes of the male antennal and eye-size characters is mapped onto the tree (Figure 6). The single most likely changes in ancestral states of the eye size character are shown with changes in branch colour from black to red or to blue (or vice versa for reversals). The single most likely changes in ancestral states of the antennal length character are shown with black (increases) or white (reversals) spots on branches. For the group with the ancestral male enlarged eye state (in red, subgenera *Mendacibombus*, *Bombias*), the largest male eye is shown by *B. nevadensis* and the smallest male eye accepted as enlarged is shown by *B. superbus*. For the group with the derived male enlarged eye state (in red or blue, in the subgenera *Sibiricobombus*, *Cullumanobombus*, *Melanobombus*), the largest male eye is shown by *B. fraternus* and the smallest male eye accepted as enlarged is shown by *B. rufocinctus*.

## 4. Discussion

### 4.1. How Does the New Tree Contribute?

The shape of the evolutionary tree for bumblebees and even its age have been understood in very broad terms for more than a century [79]. Unsurprisingly, recent molecular evidence has added many refinements [6,12,22]. Our study in turn adds more than a quarter of the world’s bumblebee species (84 species, 29%). This now covers all of the published and extant species that are currently accepted, including many that are difficult to find, to identify, and to sequence (e.g., rare species of the high mountain subgenus *Mendacibombus* [17] that are difficult to access in the remote high Asian mountains) making it taxonomically complete. It also uses some of the best tree-estimation techniques that are currently available [66,80]. Our tree (Figure 1) in many respects matches other recent estimates [12,22,58,59], but now with all species and broad genomic support, it improves estimates of relationships among some subgenera and provides greater confidence in our understanding of evolutionary relationships among all bumblebees. No doubt some problems remain, caused by (for example) paralogous copies of some genes [38,81]. Based upon all available evidence, future studies on bumblebee evolution will most likely converge upon a closely similar set of relationships.

The new evidence is contributed primarily for the relationships of intermediate age and for the more recent relationships. The older branching patterns among subgenera are supported primarily by the same four nuclear genes that largely reproduce the results of Cameron et al. [12], although where these differ the genomic results of Sun et al. [22] contribute. The subgenera used here had already been diagnosed so as to be monophyletic in the results of the Cameron et al. tree [23] and remain unchanged. At the intermediate levels of species groups within subgenera there is some original resolution that is contributed by new data from all six genes. The strongest original contribution is made among the species within the most recent species groups, contributed from the two fast-evolving mitochondrial genes, but primarily from including many more of the species (terminals on this tree).

### 4.2. Diversification

The apparent strongest and most significant reduction in the net diversification rate occurs over the last 1 Ma (Figure 3) and this has been explained previously primarily in terms of a recently greatly increased extinction rate [82]. Three causal factors were suggested: (1) rapid climatic changes; (2) human impacts such as agriculture; and (3) taxonomic uncertainty.

However, we find little evidence for apparently very young species of bumblebees in Figure 2, with only one case (the divergence between *B. validus* and *B. nobilis*) of species apparently much younger than 1 Ma. Not all processes involved in speciation may be either simultaneous or rapid [32,33]. Consequently the rarity of evidence for very young bumblebee species raises another possibility: that often it might take at least this long for species’ gene coalescents and substantial morphological differences to become detectable. These criteria have become commonly used in taxonomic revisions when recognising bumblebee species defined (as here) as evolutionarily independent lineages (Section 2.1). This might provide another explanation for some of the turn down in the tail of Figure 3.

Patterns in diversification are potentially complicated to discern from trees and all of the popular tools have faced challenges [72,83], so further developments can be expected. Nonetheless, two bumblebee groups on this tree (Figure 2: A, B) appear to show especially prominent bursts of rapid net speciation (for extant species with multiple consecutive short internode branches) that invite comment. Both of these groups are familiar for their taxonomic problems, with the constituent species being difficult to distinguish: (A) within the subgenus *Thoracobombus*, in the *fervidus*-group [7,84]; and (B) within the subgenus *Bombus* s. str., in the *lucorum*-group [36].

The map in Figure 4 shows that the highest speciation rates as measured by the sum of inverse internode lengths for bumblebee are concentrated in the faunas of Mesoamerica and South America. These high scores in the Americas are contributed especially by many species of the subgenus *Thoracobombus* with short internodes (Figure 2). This is consistent with the idea of a relatively recent arrival of these lineages, colonising a diversity of Mesoamerican and South American habitats that had previously been without bumblebees, which is likely to have driven the high speciation rates. This is an intriguing case. Some of this speciation among the currently South American lineages took place before (as dated in Figure 2) a land route for dispersal to South America became available at ca 3 Ma [85]. But bumblebees are very poor dispersers and establishers across oceans [7], so this rapid speciation must have taken place earlier within Mesoamerica with its complex high mountain ranges. These lineages must then since have become extirpated in Mesoamerica, perhaps caused by aridification from climate change [7]. Therefore some of the rapid speciation indicated on the map in Figure 4 for South American faunas is actually likely to have taken place within Mesoamerica.

None of these approaches to diversification explicitly includes the possible effects of varying extinction rates.

### 4.3. Character Evolution

The dated evolutionary tree (Figure 2) provides an improved framework for comparing the evolution of characters or traits (character states).

The greatest character-state divergence on the tree (aside from the parasitic or ‘cuckoo’ bumblebees of the subgenus *Psithyrus* that have a fundamentally different way of life and lack a worker caste [3,86], marked on Figure 1 with black spots) may be at the oldest node: between the subgenus *Mendacibombus* and the remaining extant bumblebees. This is because the bumblebees of *Mendacibombus* differ most strongly in morphology (e.g., in the form of the female labrum and the hind tibial disc bristles, and of the male genitalia) and in behaviour (e.g., in the extreme alpine specialization of all species; nests built with the nectar and pollen stores held outside the brood nest envelope, sometimes in hexagonal cells) from the other non-parasitic bumblebees [17,23,87]. These tibial bristles and the nest structures are shared with some species of the sister group to bumblebees, the stingless bees (tribe Meliponini) [88,89].

Otherwise, one of the most significant divergences is between the two larger monophyletic groups: first (Figure 1: large green spot LT/LG; divergence date estimated in Figure 2 as 24.3 Ma ± 4.9 Myr from the 95% of highest posterior density) the predominantly long-tongued [1], long-faced [12], and *Lowland Grassland* bumblebees [8]; and second (Figure 1: large blue spot ST/MG) the predominantly short-tongued [1], short-faced [12], and *Montane Grassland* bumblebees [8]. Tongue (=proboscis) length (associated with face (=malar area) length [90,91]) is important because it governs flower choice and hence diet [92]. These two large groups were once named as the ‘sections’ *Odontobombus* and *Anodontobombus* [93], although because sections no longer have nomenclatural status [94] the names have had to be synonymised with the subgeneric names *Megabombus* and *Pyrobombus* respectively [95]. These two large groups also differ in other important aspects of ecology, including broad trends: (1) towards preferring either surface or underground nesting sites respectively; (2) towards either early emergence and short colony development or late emergence and long colony development respectively; and (3) towards either cryptic or aposematic colour patterns of the hair [7,8] respectively (exceptions occur in both groups). Some of the shortest-tongued bumblebees are also the most active initiators of flower robbing [39].

### 4.4. Sexual Dimorphism and Male Mate-Searching Behaviour

Many other characters, or suites of characters, can be mapped onto the tree even when they do not associate closely with just one monophyletic group, thereby showing evidence of evolutionary convergence or parallelism. For example, while most male bumblebees have eyes of similar size to those of females (Figure 5b) and patrol circuits of pheromone-marked sites in search of females (females are presumably arrested by the male pheromones deposited at these sites so that they can be mated by returning males) [3,35,96,97], some bumblebee species (17% of species listed here) have males with either moderately or greatly enlarged eyes and pursue females in rapid flight [2,35,87,98,99,100] (Figure 5a,c). Cases of enlarged male eyes have evolved repeatedly for separate groups of bumblebees and are most likely to be the result of an ancestral state as well as multiple derived cases of parallel evolution [2,89,101,102] (Figure 6).

Enlarged male eyes for bumblebees are associated with a strong shift in male mate-searching behaviour, towards either visual scramble competition (‘racing’, associated with a combination of large eyes and long antennae, described in more detail for *B.* (*Sibiricobombus*) *longiceps*), or true territoriality resulting in aggressive displacement of competitors from symbolic (not resource-based) territories (‘lekking’, associated with a combination of large eyes and short antennae, described in more detail for *B.* (*Melanobombus*) *rufofasciatus*) [35]. Truly territorial males (high speed chases are necessary but not sufficient to characterize these) often show a higher incidence of physical damage (often with loss of parts of limbs or antennae) but crucially shows frequent replacement (turnover) of individuals on particular perches [35]. Figure 6 shows (in blue with terminal squares) that a combination for males of enlarged eyes with long antennae (8% species) characterizes most species of the subgenera *Sibiricobombus* and many species of *Cullumanobombus*, from which we expect these species to show ‘racing’ behaviour with scramble competition. Figure 6 also shows (in red with no terminal squares) that a combination for males of enlarged eyes with short antennae (9% species) characterizes species of *Mendacibombus*, *Bombias*, as well as for the species *B. morawitzi*, the *rubicundus*-group, *B. kashmirensis*, *B. rainai*, and several other species of *Melanobombus*, from which we expect these species to show true territorial behaviour with exclusion of competitors. Observations of several other of these species in Asia, Europe, and North America appear to be consistent with this pattern (e.g., [17,29,37]).

However, much detailed work remains to be done, to assess whether the association of these character states with these behaviours is truly a generality. More work is also needed to assess a proposed explanation that relates the likely relative advantages of these behaviours with predictable differences in the expected density and dispersion of receptive females in different habitats ([35] his Tables 2–4). These behaviours can also prove remarkably flexible in different circumstances for the same species [35]. Therefore we suggest that these behaviours are more likely to be secondary responses to differences in the structure of the habitats with which particular species have come to be associated (for other reasons) rather than imposing constraints directly on which habitats they can occupy.

### 4.5. Caveats and Future Work

There are many potential sources of error in estimating evolutionary trees for species from gene sequences. These include both basic problems in sampling (misidentification of samples, failing to recognise species correctly, contamination during sequencing, comparison of non-homologous sequences and alignments, incomplete lineage sorting, etc.) and problems in computation of molecular data (homoplasy, errors in specification of evolutionary models, failures in searches of possible alternative tree topologies, etc.) [80]. Our analysis is founded on a research programme of more than 40 years’ duration that has sought to reduce the effects of these problems in bumblebee studies (e.g., [12,37,38,89]). For example, with only two fast-evolving genes in our analysis, the possibilities for some misleading estimates of the more recent relationships arising from incomplete lineage sorting must be considered. This problem should be detectable in future studies when those studies can include broader genomic coverage.

One of the least certain aspects remains the dating of events on evolutionary trees. There are few informative bumblebee fossils available for the calibration of models of DNA-sequence evolution, so the precision of these estimates remains low and the implications of the uncertainty should continue to be re-examined [6,65].

We anticipate broader use of genomic data for estimating evolutionary trees for bumblebees in future studies. It will be interesting to see how those estimates compare with ours: a more extensive sampling of slow-evolving nuclear genes in genomic data should yield a more confident estimate for the older parts of the tree; it is less clear how well more broad genomic data will recover the more recent relationships, for which fast-evolving mitochondrial genes are pre-eminently suitable.

Our tree estimate is the first study to represent all of the bumblebee species world-wide. No doubt at least a few more bumblebee species will be discovered, while other taxa already known will be recognized as separate species as species’ concepts or definitions change and more data become available.

## Data Availability

Gene-sequence identifiers are listed in the Supplementary Materials file.

## Supplementary Materials

The following supporting information can be downloaded at: https://www.mdpi.com/article/10.3390/insects17060540/s1, Table S1: List of extant accepted bumblebee species world-wide with sequence identifiers; Table S2: List of the seed species and sequences used to obtain target-taxon sequences with MitoBim.

## Abbreviations

The following abbreviations are used in this manuscript:

COI	Cytochrome *c* oxidase subunit 1
DNA	Deoxyribonucleic acid
EIL	Evolutionarily independent lineages
GTR + Γ + I	General time-reversible model with gamma frequency distribution and invariant sites
MCMC	Markov chain Monte Carlo algorithm
NHMUK	Natural History Museum, United Kingdom
RNA	Ribonucleic acid
XML	Extensible mark-up language
