# Supplementary material for: Evolutionary Tree for All Bumblebee Species World-Wide Estimated by Combining Information from Fast-Evolving Genes, Slow-Evolving Genes, and Genomic Data (Apidae, Bombus)
Source: Insects. 2026 May 22;17(6):540. doi: 10.3390/insects17060540 (PMC13300377; doi:10.3390/insects17060540)
Supplement: Supplementary file 1 [file insects-17-00540-s001.zip › Supplementary Table S1.pdf]

Supplementary Table S1

List of extant accepted bumblebee species world-wide (the first three entries are outgroups and data include the possibly extinct *B. franklini;* species accepted as evolutionarily independent lineages from integrative assessment) with sequence identifiers for six genes from NCBS-GenBank and CCDB-BOLD. Names not in this list are interpreted as synonyms. Numbers in the title row give nucleotide positions in the concatenated data.

Genus/ Subgenus	Species	Author, date	16S (658-1230)	EF1-a (1231-2155)	Opsin (2156-2920)	ArgK (2921-3995)	PEPCK (3996-5420)	COI (1-657)
Eulaema	*boliviensis*	(Friese, 1898)	DQ788139	DQ788307	DQ788387	DQ788523	EF051029	EU421493
Geniotrigona	*thoracica*	Smith, 1857	DQ788140	DQ788308	DQ788388	DQ788524	EF051023	OK480965
Plebeia	*frontalis*	(Friese, 1911)	DQ790459	DQ813138	DQ813216	DQ813059	EF051027	BOLD-ASINH740-12
Mendacibombus	*superbus*	(Tkalců, 1968)	KX452097		KX452101		KX452104	IAR-HJ6
	waltoni	Cockerell, 1910	DQ788134	DQ788302	DQ788385	DQ788519	EF051022	BOLD-1550D06
	convexus	Wang, 1980	DQ787993	DQ788174	DQ788325	DQ788415	EF051021	BOLD-1550C12
	makarjini	Skorikov, 1910						BOLD-KY73
	avinoviellus	(Skorikov, 1914)	AY268416	DQ788155	AY268394	DQ788400	EF051020	BOLD-1552D05
	marussinus	Skorikov, 1910						BOLD-19992G06
	turkestanicus	Skorikov, 1910	KX452098		KX452102		KX452105	BOLD-1549C02
	defector	Skorikov, 1910	KX452099		KX452103		KX452106	BOLD-6879G08
	himalayanus	(Skorikov, 1914)						BOLD-6876D08
	margreiteri	Skorikov, 1910	KX452100	AF492958	AF493025	AF492891	KX452107	BOLD-9808D12
	handlirschianus	Vogt, 1909	DQ788022	DQ788198	DQ788337	DQ788436	EF051017	BOLD-1549E10
	mendax	Gerstaecker, 1869	DQ788057	AY739584	AF493024	AF492890	EF051019	BOLD-6876B06
Confusibombus	*confusus*	Schenck, 1859	DQ787992	DQ788173	DQ788324	DQ788414	EF051016	BOLD-BCZSMHYM09157
	nevadensis	Cresson, 1874	DQ788069	DQ788243	DQ788352	DQ788471	EF051015	BOLD-3742B11
	auricomus	(Robertson, 1903)	DQ787972	DQ788154	AY739454	AF492892	EF051014	KM585615
Kallobombus	*soroeensis*	(Fabricius, 1776)	DQ788107	DQ788278	AF493008	AF492874	EF051013	BOLD-1550E10
Subterraneobombus	*fedtschenkoi*	Morawitz in Fedtschenko, 1875						NHM-ADL-MW06
	personatus	Smith, 1879	DQ788081	DQ788256	DQ788359	DQ788481	EF050993	BOLD-ST3445
	tschitscherini	Radoszkowski, 1862						BOLD-ST3824
	melanurus	Lepeletier de Saint Fargeau, 1835	DQ788056	DQ788230	AF492990	AF492856	EF050991	BOLD-ST1864
	difficillimus	Skorikov, 1912	DQ787998	DQ788177	DQ788327	DQ788417	EF050990	BOLD-ST3446
	mongolensis	Williams in Williams *et al.*, 2011						BOLD-ST1939
	amurensis	Radoszkowski, 1862						BOLD-ST1970
	fragrans	(Pallas, 1771)	DQ788012	DQ788191	DQ788333	DQ788430	EF050992	BOLD-ST2523
	subterraneus	(Linnaeus, 1758)	DQ788109	DQ788281	AF493027	AF492893	EF050989	BOLD-ST3359
	distinguendus	Morawitz, 1869	DQ787999	DQ788180	DQ788328	DQ788420	EF050988	BOLD-PCHELAB12
	appositus	Cresson, 1878	DQ787965	DQ788148	DQ788314	DQ788395	EF050986	BOLD-ST271
	borealis	Kirby, 1837	DQ787981	AF492976	AF493043	AF492909	EF050987	BOLD-ST255
Megabombus	*diversus*	Smith, 1869	DQ788000	AF492961	AF493028	AF492894	EF051010	KP671669
	ussurensis	Radoszkowski, 1877	AF364829	AF492919	AF492986	AF492852	EF051008	KP671675
	longipes	Friese, 1905	DQ788049	DQ788223	DQ788346	DQ788455	EF051009	KP671681
	montivagus	Smith, 1878						KP671648
	albopleuralis	Friese, 1916	DQ788122	AF492918	AF492985	AF492851	EF051007	BOLD-1550C02
	burmensis	Williams, 2020						KP671668
	trifasciatus	Smith, 1852	DQ788135	KX791652	KX791678	KX791727	KX791702	KP671661

	bicoloratus	Smith, 1879	DQ787976	AF492971	AF493038	AF492904	EF051005	IAR-BI00006
	irisanensis	Cockerell, 1910						CT-1011
	senex	Snellen van Vollenhoven, 1873						NHM-ADL-MW03
	supremus	Morawitz, 1887	DQ788112	DQ788284	DQ788375	DQ788505	EF051004	IAR-SU00013
	gerstaeckeri	Morawitz, 1881	DQ788017	DQ788195	DQ788335	DQ788434	EF051003	BOLD-FBHAP779-09
	argillaceus	(Scopoli, 1763)	DQ787967	DQ788150	AY739453	DQ788396	EF051001	IAR-murat2-20
	ruderatus	(Fabricius, 1775)	DQ788096	AF492977	AF493044	AF492910	EF051002	IAR-paulF5
	portchinsky	Radoszkowski, 1884	DQ788085	DQ788259	DQ788361	DQ788484	EF051000	IAR-murat18
	hortorum	(Linnaeus, 1761)	DQ788024	DQ788200	AF492987	AF492853	EF050999	IAR-murat2
	securus	(Frison, 1935)	DQ788100	DQ788270	DQ788368	DQ788493	EF050997	IAR-SE0078
	religiosus	(Frison, 1935)	DQ788091	DQ788264	DQ788364	DQ788489	EF050998	IAR-RE00008
	czerskii	Skorikov, 1910						IAR-CZ00024
	tichenkoi	(Skorikov, 1926)		AF492922	AF492989	AF492855		BOLD-1550H11
	consobrinus	Dahlbom, 1832	EF032354	EF032379	AY267150	AY267166	EF050994	IAR-CO00163
	koreanus	(Skorikov, 1933)	EF032355	AF492969	AF493036	AF492902	EF050995	IAR-KO00041
Orientalibombus	*braccatus*	Friese, 1905						BOLD-1552A03
	funerarius	Smith, 1852	EF032356	EF032378	EF032396	EF032415	EF051012	FJ175342
	haemorrhoidalis	Smith, 1852	DQ788020	AF492935	AF493002	AF492868	EF051011	BOLD-3261H04
Thoracobombus	*dahlbomii*	Guérin-Méneville, 1835	DQ787996	AF492931	AF492998	AF492864	EF050940	EF-PauloBarBeef
	excellens	Smith, 1879	AY268405	DQ788184	AY268383	DQ788424	EF050942	KC853321
	morio	(Swederus, 1787)	AY268409	DQ788239	AY268387	DQ788467	EF050941	EF-Jaboticatuba
	pomorum	(Panzer, 1805)	DQ788084	DQ788258	DQ788360	DQ788483	EF050939	UM-BM1
	mesomelas	Gerstaecker, 1869	DQ788058	DQ788231	EF032390	EF032414	EF050938	BOLD-563805
	armeniacus	Radoszkowski, 1877	DQ787968	DQ788151	DQ788315	DQ788397	EF050937	BOLD-KY127
	laesus	Morawitz, 1875	DQ788044	DQ788218	DQ788344	DQ788451	EF050936	BOLD-SHMELG06
	mucidus	Gerstaecker, 1869	DQ788066	AF492935	AF493002	AF492868	EF050934	BOLD-NBRA0352
	persicus	Radoszkowski, 1881	DQ788080	DQ788255	DQ788358	DQ788480	EF050935	BOLD-NBRA1050
	zonatus	Smith, 1854	DQ788138	DQ788306	DQ788386	DQ788522	EF050926	UM-BM2
	anachoreta	(Skorikov, 1914)						AF279548
	deuteronymus	Schultz, 1906	DQ787997	DQ788176	DQ788326	AY267170	EF050922	BOLD-PCHELAC05
	humilis	Illiger, 1806	DQ788026	DQ788202	AY739469	DQ788439	EF050924	ZR-GZM101004
	filchnerae	Vogt, 1908	DQ788008	DQ788188	DQ788331	DQ788428	EF050933	ZR-GZM104002
	muscorum	(Linnaeus, 1758)	DQ788067	DQ788241	DQ788351	DQ788469	EF050925	BOLD-KY261
	velox	(Skorikov, 1914)	DQ788127	DQ788296	DQ788381	DQ788514	EF050921	UM-N48
	ruderarius	(Müller, 1776)	DQ788095	AF492932	AF492999	AF492865	EF050920	GU705935
	veteranus	(Fabricius, 1793)	DQ788129	DQ788298	DQ788382	DQ788516	EF050919	BOLD-KY266
	inexspectatus	(Tkalců, 1963)						UM-N261
	mlokosievitzii	Radoszkowski, 1877	DQ788061	DQ788235	DQ788349	DQ788463	EF050917	UM-N44
	sylvarum	(Linnaeus, 1761)	DQ788114	DQ788285	DQ788376	DQ788506	EF050918	BOLD-00610H02
	pascuorum	(Scopoli, 1763)	DQ788077	DQ788251	AF493001	AY492867	EF050932	BOLD-00610H04
	remotus	(Tkalců, 1968)	DQ788092	DQ788265	DQ788365	DQ788490	EF050929	ZR-2018053
	impetuosus	Smith, 1871	EF032350	EF032373	EF032391	EF032409	EF050927	ZR-ZLSM303002
	honshuensis	(Tkalců, 1968)		AF493029	AF492962	AF492895		AF279559
	opulentus	Smith, 1861	KX791786	KX791660	KX791684	KX791734	KX791710	KX791760
	schrencki	Morawitz, 1881	AF364828	AF492933	AF493000	AF492866	EF050931	BOLD-1550F03
	pseudobaicalensis	Vogt, 1911	EF032357	EF032372	AY267155	AY267171	EF050923	AF279562
	hedini	Bischoff, 1936	DQ788023	DQ788199	DQ788338	DQ788437	EF050930	ZR-HED4
	imitator	Pittioni, 1949	DQ788032	DQ788208	DQ788339	DQ788441	EF050965	IAR-JHIM01
	exil	(Skorikov, 1923)	DQ788004	DQ788185	DQ788330	DQ788425	EF050962	BOLD-9808E10

	tricornis	Radoszkowski, 1888	DQ788121	AF492937	AF493004	AF492870	EF050963	AF279573
	atripes	Smith, 1852	DQ787971	DQ788153	DQ788316	DQ788399	EF050964	IAR-JHAT01
	digressus	(Milliron, 1962)	AY268403	DQ788178	AY268381	DQ788418	EF050959	ECOAB-66857
	weisi	Friese, 1903	AY268415	DQ788303	AY268393	DQ788520	EF050961	ECOAB-9555
	nigrodorsalis	Franklin, 1907						ECOAB-200736
	trinominatus	von Dalla Torre, 1890	DQ788123	DQ788292	DQ788379	DQ788511	EF050960	ECOAB-16124
	fervidus	(Fabricius, 1798)	AY268406	AF492930	AF492997	AF492863	EF050958	BOLD-3759F04
	diligens	Smith, 1861	AY268404	DQ788179	AY268382	DQ788419	EF050955	ECOAB-323
	opifex	Smith, 1879	DQ788075	DQ788249	DQ788356	DQ788477	EF050954	KC853365
	bellicosus	Smith, 1879	AY268399	DQ788159	AY268377	DQ788404	EF050956	EF-SCSaoJoaquim
	medius	Cresson, 1863	AY268407	DQ788228	AY268385	DQ788459	EF050951	ECOAB-74419
	steindachneri	Handlirsch, 1888	AY268413	DQ788280	AY268391	DQ788502	EF050953	ECOAB-16095
	mexicanus	Cresson, 1878	AY268408	DQ788232	AY268386	DQ788461	EF050952	ECOAB-79260
	pensylvanicus	(DeGeer, 1773)	EF032352	EF032374	EF032392	EF032411	EF050948	BOLD-07NM2117
	pullatus	Franklin, 1913	AY268411	DQ788261	AY268389	DQ788486	EF050945	ECOAB-10467
	brevivillus	Franklin, 1913	OM649804	OM649766	OM649773	OM649782	OM649792	EF-PA.Oriximina
	pauloensis	Friese, 1913	AY268398	DQ788152	AY268376	DQ788398	EF050943	EF-SP.Guaratuba
	transversalis	(Olivier, 1789)	AY268414	DQ788291	AY268392	DQ78810	EF050944	A3051S37
	brasiliensis	Lepeletier de Saint Fargeau, 1835	AY268400	DQ788164	AY268378	OM649781	EF050950	EF-FOFBigu4.SC.Biguasu
	bahiensis	Santos-Junior, Santos & Silveira, 2015	OM649806	OM649763	OM649771	OM649780	OM649788	KJ848944
Psithyrus	*citrinus*	(Smith, 1854)	DQ787990	DQ788171	DQ788322	AY267169	EF050975	FJ582111
	insularis	(Smith, 1861)	DQ788038	AF492975	AF493042	AF492908	EF050976	HQ554083
	intrudens	(Smith, 1861)	AY268419	DQ788295	AY268397	DQ788513	EF050977	ECOAB-BUMB0361
	morawitzianus	(Popov, 1931)						BOLD-KY275
	campestris	(Panzer, 1801)	DQ787986	AF492927	AF492994	AF492860	EF050974	BOLD-BCZSMHYM01054
	ferganicus	(Radoszkowski, 1893)						NHM-ML-1
	bellardii	(Gribodo, 1891)						BOLD-B1397H05
	turneri	(Richards, 1929)	KX791779	KX791653		KX791728	KX791703	KX791753
	tibetanus	(Morawitz, 1887)	DQ788120	DQ788290	DQ788378	DQ788509	EF050985	ZR-AJLSH201044
	cornutus	(Frison, 1933)	EF032353	EF032377	EF032395	EF032413	EF050984	BOLD-1551E09
	expolitus	(Tkalců, 1989)						ZR-ZLSM301004
	novus	(Frison, 1933)						NHM-ADL-LR59
	chinensis	(Morawitz, 1890)	DQ787988	DQ788170	DQ788321	DQ788412	EF050982	ZR-41.XL3.2018.6.18
	branickii	(Radoszkowski, 1893)						ZR-AJLSH101050
	rupestris	(Fabricius, 1793)	DQ788099	AF492928	AF492995	AF492861	EF050983	GU674497
	suckleyi	Greene, 1860	DQ788110	DQ788282	DQ788374	DQ788503	EF050981	BOLD-15289C03
	vestalis	(Geoffroy in Fourcroy, 1785)	DQ788128	DQ788297	AY739495	DQ788515	EF050980	BOLD-BCZSMHYM01140
	bohemicus	Seidl, 1837	DQ787980	AF492925	AF492992	AF492858	EF050979	ZR-GLLSM201075
	coreanus	(Yasumatsu, 1934)	AF364830					IAR-3C02BN17
	barbutellus	(Kirby, 1802)	DQ787975	DQ788158	DQ788318	DQ788403	EF050972	BOLD-BCZSMHYM01047
	sylvestris	(Lepeletier de Saint Fargeau, 1832)	DQ788115	DQ788286	DQ788377	DQ78807	EF050967	GU694197
	quadricolor	(Lepeletier de Saint Fargeau, 1832)	DQ788090	DQ788263	DQ788363	DQ788488	EF050966	GU694182
	norvegicus	(Sparre-Schneider, 1917)	DQ788072	DQ788246	DQ788354	DQ788474	EF050971	GU694186
	flavidus	Eversmann, 1852	DQ788006	AF492926	AF492993	AF492859	EF050969	BOLD-07-ID-1429

	skorikovi	(Popov, 1927)	DQ788106	DQ788276	DQ788373	DQ788499	EF050968	ZR-YLSH101042
Pyrobombus	*vagans*	Smith, 1854	DQ788125	DQ788293	DQ788380	DQ788512	FE050854	BOLD-3746B07
	caliginosus	(Frison, 1927)	DQ787985	DQ788168	AF493035	AF492901	EF050853	BOLD-97CA2615
	centralis	Cresson, 1864	DQ787987	DQ788169	AY739459	DQ788411	EF050852	BOLD-UAIC1135108
	flavifrons	Cresson, 1863	DQ788011	DQ78810	AF493016	AF492882	EF050850	BOLD-JSYKB084
	vandykei	(Frison, 1927)	DQ788126	DQ788294	AF493049	AF492915	EF050851	BOLD-LRBBC1588
	melanopygus	Nylander, 1848	DQ788055	DQ788229	AF493011	AF492877	EF050849	BOLD-10BBCHY3180
	bimaculatus	Cresson, 1863	DQ787978	DQ788161	AY739456	DQ788405	EF050847	KM585629
	konradini	Reinig, 1965						BOLD-ULB004
	lapponicus	(Fabricius, 1793)	DQ788046	DQ788220	DQ788345	DQ788452	EF050845	BOLD-KY295
	glacialis	Friese, 1902						MK530673
	johanseni	Sladen, 1919						BOLD-38772G05
	monticola	Smith, 1849	DQ788064	DQ788238	AY739483	DQ788466	EF050848	NHM-BMNH970373
	huntii	Greene, 1860	DQ788027	DQ788203	AF493045	AF492911	EF050840	BOLD-BIOUG02462H06
	vosnesenskii	Radoszkowski, 1862	DQ788133	DQ788301	AF493047	AF492913	EF050841	BOLD-10BBCHY3209
	ternarius	Say, 1837	DQ788117	AF492979K	AF493046	AF492912	EF050839	BOLD-10BBCHY3178
	bifarius	Cresson, 1878	DQ787977	DQ788160	AF493010	AF492876	EF050838	BOLD-03760H03
	impatiens	Cresson, 1863	DQ788033	DQ788209	AF493009	AF492875	EF050842	BOLD-BIOUG00835A02
	ephippiatus	Say, 1837	DQ788002	DQ788182	AY739462H	DQ788422	EF050844	AQ118451
	haematurus	Kriechbaumer, 1870	DQ788019	EF032364			EF050829	BOLD-ZPLBI00922
	wolongensis	Williams, Ren & Xie, 2022						BOLD-596G12
	bryorum	Richards, 1930						BOLD-1550N539
	taiwanensis	Williams, Sung, Lin & Lu, 2022						MZ831884
	hypnorum	(Linnaeus, 1758)	DQ788029	DQ788205	AF493013	AF492879	EF050826	BOLD-1550A09
	koropokkrus	Sakagami & Ishikawa, 1972						HQ553056
	hengduanensis	Williams, Ren & Xie, 2022	EF032359	EF032363	EF032383	EF032401	EF050827	BOLD-1550A04
	perplexus	Cresson, 1863	DQ788079	DQ788254	AF493012	AF492878	EF050828	BOLD-1550H03
	subtypicus	(Skorikov, 1914)						BOLD-1551D05
	biroi	Vogt, 1911	DQ787979	DQ788162	AY739457	DQ788406	EF050825	BOLD-KY327
	nursei	Friese, 1918						BOLD-6879H11
	kotzschi	Reinig, 1940						NHM-ADL-MW05
	ardens	Smith, 1879	DQ787966	DQ788149	AF493031	AF492897	EF050823	MF361563
	flavescens	Smith, 1852	DQ788009	AF492950	AF493017	AF492883	EF050824	ZR-XL22018625
	rotundiceps	Friese, 1916						BOLD-182Q09
	pyrenaeus	Pérez, 1879	DQ788089	DQ788262	AY739488	DQ788487	EF050822	GU705926
	wangae	Williams, Tang, Yao & Cameron, 2009	DQ788063	DQ788237	AY739482H	DQ788465	EF050820	BOLD-3262D07
	modestus	Eversmann, 1852	EF032358	EF032362	EF032382	EF032400	EF050821	BOLD-PCHEL025-09
	beaticola	(Tkalců, 1968)		AF492963	AF493030	AF492896		AF385813
	brodmannicus	Vogt, 1909	DQ787984	DQ788166	AY739458	DQ788409	EF050818	BOLD-1551H04
	pratorum	(Linnaeus, 1761)	DQ788087	AF492966	AF493033	AF492899	EF050819	BOLD-BCZSMHYM01109
	cingulatus	Wahlberg, 1854	DQ787989	AF492948	AF493015	AF492881	EF050812	BOLD-FISYM011-14
	jonellus	(Kirby, 1802)	DQ788039	DQ788214	AY739473	DQ788446	EF050814	BOLD-15289A08
	frigidus	Smith, 1854	DQ788014	DQ788192	AY739466	DQ788431	EF050811	BOLD-CHU06BEE033
	sitkensis	Nylander, 1848	DQ788105	DQ788275	AY739490	DQ788498	EF050817	BOLD-LRBBC1622
	mixtus	Cresson, 1878	DQ788060	DQ788234	AF493014	AF492880	EF050816	BOLD-04485G09
	sandersoni	Franklin, 1913						BOLD-04485D10WI
	infirmus	(Tkalců, 1968)	DQ788036	DQ788212	AY739471	DQ788444	EF050836	NBC10112
	pressus	(Frison, 1935)	DQ788088	EF032368			EF050837	IOZ-BB05
	abnormis	(Tkalců, 1968)						BOLD-1550B08
	semiperalpinus	(Tkalců, 1974)						BOLD-1555F02
	lemniscatus	Skorikov, 1912	DQ788047	DQ788221	AY739477H	DQ788453	EF050834	ZR-DQM101002
	lepidus	Skorikov, 1912	DQ788048	DQ788222	AY739478H	DQ788454	EF050835	ZR-DQM101012

	mirus	(Tkalců, 1968)						IAR-BB04
	picipes	Richards, 1934	DQ788082	DQ788257	AY739487H	DQ788482	EF050833	IAR-NX9
	luteipes	Richards, 1934	DQ788052					BOLD-6877D07
	avanus	(Skorikov, 1937)	EF032344	EF032365	EF023384	EF032402	EF050830	BOLD-6880F03
	infrequens	(Tkalců, 1989)	DQ788037	DQ788213	AY739472H	DQ788445	EF050831	BOLD-6877D02
	parthenius	Richards, 1934	DQ788076	DQ788250	DQ788357	DQ788478	EF050832	BOLD-1551F05
	sonani	(Frison, 1934)		AF492951	AF493018	AF492884		NHM-ML-P018
Bombus	*sporadicus*	Nylander, 1848	DQ788108	DQ788279	AY739491	DQ788501	EF050867	BOLD-6876D06
	czerskianus	Vogt, 1911						BOLD-6875C09
	ignitus	Smith, 1869	DQ788031	DQ788207	AF493032	AF492898	EF050866	BOLD-6878F06
	terrestris	(Linnaeus, 1758)	DQ788118	DQ788288	AF493022	AF492888	EF050865	BOLD-6878G03
	xanthopus	Kriechbaumer, 1870						BOLD-6879B05
	tunicatus	Smith, 1852	DQ788124					BOLD-1551G11
	jacobsoni	Skorikov, 1912						BOLD-6878B02
	hypocrita	Pérez, 1905	DQ788030	DQ788206	AF493023	AF492889	EF050864	NC011923
	reinigi	Tkalců, 1974						BOLD-0110NEP
	longipennis	Friese, 1918						IAR-LU247
	minshanicola	Bischoff, 1936	DQ788050	DQ788224	AY739479	DQ788456	EF050863	IAR-HJLU248
	affinis	Cresson, 1863	DQ787961	DQ788144	AY739451	DQ788391	EF050860	BOLD-3742G12
	franklini†	(Frison, 1921)	EF032345	EF032366	EF032385	EF032403	EF050861	AY694097
	lucorum	(Linnaeus, 1761)	DQ788051	DQ788225	AF493021	AF492887	EF050862	BOLD-6875C05
	terricola	Kirby, 1837	DQ788119	DQ788289	AF493019	AF492885	EF050859	BOLD-3759F10
	occidentalis	Greene, 1858	DQ788074	DQ788248	AY739486	DQ788476	EF050858	BOLD-07CO2119
	mckayi	Ashmead, 1902						BOLD-6706D07
	cryptarum	(Fabricius, 1775)	DQ787995	DQ788175	AY739461	DQ788416	EF050855	NHM-173-T727
	patagiatus	Nylander, 1848	KX791787	KX791661	KX791685	KX791735	KX791711	BOLD-PCHELAC02
	ganjsuensis	Skorikov, 1913						BOLD-6875D12
	magnus	Vogt, 1911						BOLD-6876D09
	lantschouensis	Vogt, 1908	KX791784	KX791658	KX791682	KX791732	KX791708	NHM-5-T545
	minshanensis	Bischoff, 1936	DQ788078	DQ788252	AF493020	AF492886	EF050857	NHM-2-T542
Alpinobombus	*alpinus*	(Linnaeus, 1758)	DQ787963	DQ788146	AY739452	DQ788393	EF050871	BOLD-6873F07
	polaris	Curtis in Ross, 1835	MK511978		MK532042		KP901107	BOLD-9863C04
	pyrrhopygus	Friese, 1902	DQ788083	AF492970	AF493037	AF492903	EF050872	BOLD-20945H03
	kirbiellus	Curtis in Ross, 1835	MK511975		M532039		KP901105	BOLD-6879E12
	balteatus	Dahlbom, 1832	DQ787974	DQ788157	AY739455	DQ788402	EF050870	BOLD-NOAPI567
	neoboreus	Sladen, 1919	DQ788068	DQ788242	AY739484	DQ788470	EF050869	BOLD-15289C04
	kluanensis	Williams & Cannings, 2016	MK511976		M532040		KP901108	BOLD-20946H08
	natvigi	Richards, 1931	MK511977		MK532041		KP901106	BOLD-6879G04
	hyperboreus	Schönherr, 1809	DQ788028	DQ788204	AY739470	DQ788440	EF050868	BOLD-NOAPI569
Alpigenobombus	*wurflenii*	Radoszkowski, 1860						BOLD-1555A01
	mastrucatus	Gerstaecker, 1869	DQ788137	DQ788305	AF493007	AF492873	EF050914	AY181175
	kashmirensis	Friese, 1909	DQ788040	DQ788215	DQ788342	DQ788447	EF050913	ZR-ESM302006
	rainai	Williams, 2022						BOLD-1552E01
	sikkimi	Friese, 1918						MS-BE206
	nobilis	Friese, 1905	DQ788071	DQ788245	AY739485	DQ788473	EF050912	ZR-DDSM202013
	validus	Friese, 1905						IAR-HJ16
	genalis	Friese, 1918						IAR-093
	breviceps	Smith, 1852	DQ787983	DQ788165	DQ788320	DQ788408	EF050915	IAR-NX2
	grahami	(Frison, 1933)	EF032347	EF032371	EF032389	EF032408	EF050916	IAR-030
	angustus	Chiu, 1948						MZ831894
Melanobombus	*eximius*	Smith, 1852	DQ788005	DQ788186	AY739464	DQ788426	EF050911	BOLD-3261H06

	rufipes	Lepeletier de Saint Fargeau, 1835						BOLD-1555E02
	festivus	Smith, 1861	DQ788007	DQ788187	AY739465	DQ788427	EF050910	IOZ-MO44
	simillimus	Smith, 1852	DQ788104	DQ788274	DQ788372	DQ788497	EF050909	NHM-ML-PW18
	prshewalskyi	Morawitz, 1880	DQ788098	DQ788269	AY739489	DQ788492	EF050907	BOLD-6876H09
	rufofasciatus	Smith, 1852						BOLD-1555E09
	miniatus	Bingham, 1897						BOLD-6880H08
	eurythorax	Wang, 1982	DQ788059	DQ788233	DQ788348	DQ788462	EF050908	BOLD-6877G10
	richardsiellus	(Tkalců, 1968)						NHM-CT1
	friseanus	Skorikov, 1933	DQ788015	DQ788193	AY739467	DQ788432	EF050906	BOLD-3262F02
	pyrosoma	Morawitz, 1890	KX791774	KX791648	X791674	KX791723	KX791698	BOLD-1555C03
	formosellus	(Frison, 1934)		AF492939	AF493006	AF492872		NHM-ML-PW09
	ladakhensis	Richards, 1928	DQ788043	AY739575	AY739475	DQ788450	EF050905	BOLD-1555G10
	sichelii	Radoszkowski, 1859	DQ788103	DQ788273	DQ788371	DQ788496	EF050900	BOLD-1550G02
	tanguticus	Morawitz, 1887						NHM-ML-RC2
	incertus	Morawitz, 1881	DQ788035	DQ788211	DQ788341	DQ788443	EF050901	BOLD-1555C08
	eriophorus	Klug, 1807						BOLD-1555A04
	lapidarius	(Linnaeus, 1758)	DQ788045	DQ788219	AF493005	AF492871	EF050902	BOLD-6877H03
	semenovianus	(Skorikov, 1914)						BOLD-1555E11
	tibeticus	Williams in Williams *et al*., 2020						BOLD-6877F09
	alagesianus	Reinig, 1930	DQ787962	DQ788145	DQ788312	DQ788392	EF050903	BOLD-6876H10
	incertoides	Vogt, 1911						BOLD-6880B07
	qilianensis	Williams in Williams *et al*., 2020	DQ788041	DQ788216	AY739474	DQ788448	EF050904	BOLD-1555H05
	keriensis	Morawitz, 1887						BOLD-1555D01
	separandus	Vogt, 1909						BOLD-1555F12
Sibiricobombus	*semenovi*	Morawitz, 1887	DQ788073	DQ788247	DQ788355	DQ788475	EF050898	NHM-ML-C00
	oberti	Morawitz, 1883						NHM-ADL-K01
	morawitzi	Radoszkowski, 1876					NHM-ML-BL04	NHM-ML-C06
	sibiricus	(Fabricius, 1781)	EF032348	EF032370	EF032388	EF032407	EF050897	BOLD-6880C11
	sulfureus	Friese, 1905	DQ788111	DQ788283	AY739492	DQ788504	EF050895	NHM-ML-I027
	niveatus	Kriechbaumer, 1870	DQ788070	DQ788244	DQ788353	DQ788472	EF050893	BOLD-6880G01
	obtusus	Richards, 1951						BOLD-6880D07
	tescorum	Williams in Williams *et al*., 2026						BOLD-6880E03
	longiceps	Smith, 1878	DQ787970	EF032369	EF032387	EF032406	EF050896	BOLD-1552D10
	falsificus	Richards, 1930						IAR-BB20
	asiaticus	Morawitz in Fedtschenko, 1875						BOLD-KY665
Cullumanobombus	*rufocinctus*	Cresson, 1863	DQ788097	DQ788268	AF493034	AF492900	EF050892	BOLD-07WA1428
	cullumanus	(Kirby, 1802)	DQ787964	DQ788147	DQ788313	DQ788394	EF050890	BOLD-1551D07
	semenoviellus	Skorikov, 1910	DQ788101	DQ788271	DQ788369	DQ788494	EF050891	BOLD-6878A02
	unicus	Morawitz, 1883						BOLD-6875A07
	brachycephalus	Handlirsch, 1888	DQ787982	DQ788163	DQ788319	DQ788407	EF050889	ECOAB-BUMB0316
	haueri	Handlirsch, 1888						ECOAB-BUB0373
	rubicundus	Smith, 1854	DQ788094	DQ788267	DQ788367	DQ788491	EF050888	MK516180
	handlirschi	Friese, 1903	DQ788021	DQ788197	DQ788336	DQ788435	EF050887	PA-AHT1480
	coccineus	Friese, 1903	DQ787991	DQ788172	DQ788323	DQ788413	EF050885	
	baeri	Vachal, 1904	DQ787973	DQ788156	DQ788317	DQ788401	EF050886	
	morrisoni	Cresson, 1878	DQ788065	DQ788240	DQ788350	DQ788468	EF050880	BOLD-01UT2597
	griseocollis	(DeGeer, 1773)	DQ788018	DQ788196	AF493039	AF492905	EF050879	BOLD-LRBBC783

crotchii	Cresson, 1878	DQ787994	AF492973	AF493040	AF492906	EF050882	BOLD-08CA2051
macgregori	Labougle & Ayala, 1985	DQ788053	DQ788226	DQ788347	DQ788457	EF050881	ECOAB-BUMB0295
rohweri	(Frison, 1925)						NHM-ML-HH
funebris	Smith, 1854	DQ788016	DQ788194	DQ788334	DQ788433	EF050883	PA-A3AHS438
fraternus	(Smith, 1854)	DQ788013	AF492974	AF493041	AF492907	EF050884	BOLD-06704B04
vogti	Friese, 1903	DQ788130	DQ788299	DQ788383	DQ788517	EF050878	
ecuadorius	Meunier, 1890	DQ788001	DQ788181	DQ788329	DQ788421	EF050874	BOLD-24230A08
hortulanus	Friese, 1904	DQ788025	DQ788201	AY739468	DQ788438	EF050875	MK516188
robustus	Smith, 1854	DQ788093	DQ788266	DQ788366	EF032405	EF050877	MK516121
melaleucus	Handlirsch, 1888	DQ787960	DQ788143	DQ788311	DQ788460	EF050876	BOLD-1555D12
tucumanus	Vachal, 1904	EF032349	EF032367	EF032386	EF032404	EF050873	MA-TUC
