# Supplementary material for: Evolutionary Tree for All Bumblebee Species World-Wide Estimated by Combining Information from Fast-Evolving Genes, Slow-Evolving Genes, and Genomic Data (Apidae, Bombus)
Source: Insects. 2026 May 22;17(6):540. doi: 10.3390/insects17060540 (PMC13300377; doi:10.3390/insects17060540)
Supplement: Supplementary file 1 [file insects-17-00540-s001.zip › Supplementary Table S2.pdf]

Supplementary Table S2

List of the seed species and COI5P barcode sequences used to obtain target-taxon barcode sequences with MitoBim.

Target barcode ID	Target taxon	Seed species	Barcode seed ID
K01	*B. oberti*	*B. semenovi*	NHMMLC00
MW02	*B. melanopoda*	*B. supremus*	BK063638
MW03	*B. senex*	*B. consobrinus*	MF995069
MW04	*B. variabilis*	*B. citrinus*	BK063616
MW05	*B. kotzschi*	*B. picipes*	BK063636
MW06	*B. fedtschenkoi*	*B. difficillimus*	BK063619
LR59	*B. novus*	*B. chinensis*	ZR41.XL3.2018.6.18
